# Signaling pathway-based culture condition improves differentiation potential of canine induced pluripotent stem cells

**DOI:** 10.1016/j.stemcr.2025.102640

**Published:** 2025-09-18

**Authors:** Toshiya Nishimura, Kazuto Kimura, Kyomi J. Igarashi, Kohei Shishida, Hiroko Sugisaki, Masaya Tsukamoto, Aadhavan Balakumar, Chihiro Funamoto, Masumi Hirabayashi, Amir Kol, Shingo Hatoya

**Affiliations:** 1 Premium Research Institute for Human Metaverse Medicine (WPI-PRIMe), The University of Osaka, Suita, Osaka 565-0871, Japan; 2 Department of Advanced Pathobiology, Graduate School of Veterinary Sciences, Osaka Metropolitan University, Izumisano, Osaka 598-8531, Japan; 3 Department of Pathology, Microbiology & Immunology, School of Veterinary Medicine, University of California, Davis, Davis, CA 95616, USA; 4 Institute for Stem Cell Biology and Regenerative Medicine, Stanford University School of Medicine, Stanford, CA, USA; 5 Department of Genetics, Stanford University School of Medicine, Stanford, CA, USA; 6 Division of Stem Cell and Organoid Medicine, Department of Genome Biology, Graduate School of Medicine, The University of Osaka, Osaka 565-0871, Japan; 7 Center for Genetic Analysis of Behavior, National Institute for Physiological Sciences, Okazaki, Aichi 444-8787, Japan; 8 The Graduate University of Advanced Studies, Okazaki, Aichi 444-8787, Japan

**Keywords:** canine, induced pluripotent stem cells, cardiomyocyte, reporter line, gene editing, primed pluripotent stem cells, animal

## Abstract

Naturally occurring diseases in companion dogs are increasingly being recognized as valuable translational disease models. While induced pluripotent stem cell (iPSC) technology had revolutionized the field of human bio-medical research, canine iPSC (ciPSC) technology is still in its infancy, and robust canine-specific iPSC medium formulations and differentiation protocols are lacking. Here, we have established NANOG-reporter ciPSC lines and found that fibroblast growth factor (FGF), activin/transforming growth factor (TGF)-β, and WNT signals were critical for the robust maintenance of ciPSCs. Manipulating these signaling pathways stabilized the culture of ciPSC regardless of the cell line or basal medium. ciPSCs cultured in the optimized medium showed a homogenized global gene expression pattern. Furthermore, the ciPSCs cultured in this medium successfully differentiated into cardiomyocytes displaying homogenous contraction as well as sarcomere alignment. This robust culture condition provides a valuable resource to facilitate the utilization of ciPSCs for various studies, including human disease modeling.

## Highlights

• FGF, activin/TGF-β, and WNT signals are critical for ciPSC maintenance in culture
• Activin + WNT inhibitor (AR) medium stabilizes ciPSCs and reduces heterogeneity
• AR medium enhances ciPSC developmental potency regardless of the cell line
• AR medium enables ciPSC differentiation into cardiomyocytes with functional traits

## Graphical abstract

**Figure:**
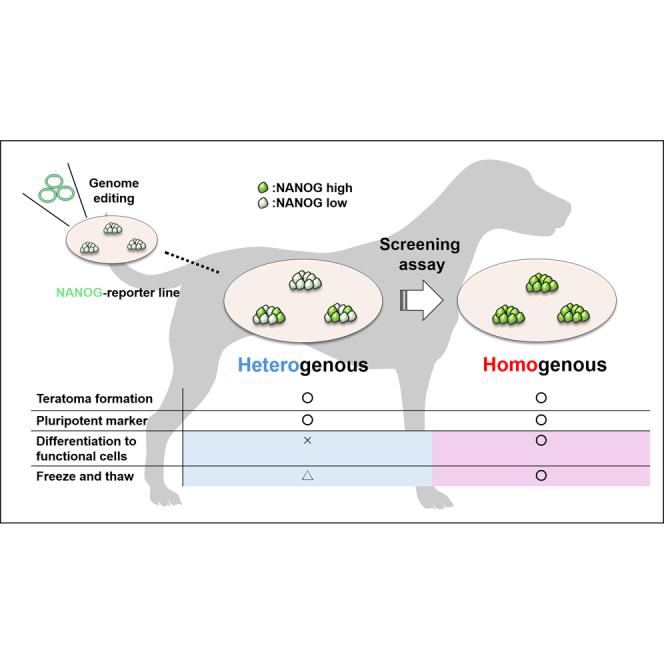

## Teaser

Nishimura and colleagues demonstrate that the modulation of activin/TGF beta and WNT signaling pathways enables the robust maintenance and stable culture of ciPSCs across multiple cell lines and basal media. Using this optimized medium, ciPSCs successfully differentiate into cardiomyocytes displaying homogenous contractions and sarcomere organization. These findings represent key steps toward overcoming long-standing barriers in ciPSC cultures and applications.

## Introduction

Pluripotent stem cells (PSCs) have unlimited proliferation capacity and the potential to differentiate into all cell types in the body. The PSCs can be categorized into two groups based on their origins: embryonic stem cells (ESCs) derived from embryos and induced PSCs (iPSCs) reprogrammed from somatic cells from which they inherit genetic signatures (Takahashi et al., 2007; Thomson et al., 1998). Therefore, iPSCs can be established from patient-derived somatic cells and used for not only developing new drugs but also individualized patient therapy (Rowe and Daley, 2019).

While all the PSCs possess pluripotency, those in different states (naive and primed) display distinct characteristics (Nichols and Smith, 2009). In mice, epiblast stem cells (EpiSCs), derived from the epiblast of mid-late gastrulation embryos, are defined as primed PSCs (Brons et al., 2007; Nichols and Smith, 2009; Tesar et al., 2007). They are cultured in media supplemented with basic fibroblast growth factor (bFGF) and activin. Primed PSCs in other species also exhibit similar characteristics to those of mouse EpiSCs. They can be distinguished from naive PSCs, which display characteristics of the earlier stage pre-implantation embryos and are therefore capable of forming chimeric animals (Iwatsuki et al., 2023; Kinoshita et al., 2021b; Kobayashi et al., 2021). The similarities among the EpiSC lines of the different species are reflected in their culture conditions, which require the same cytokines and small molecules, bFGF, activin/transforming growth factor (TGF)-β, and WNT inhibitor (Iwatsuki et al., 2023; Kinoshita et al., 2021b; Kobayashi et al., 2021). Canines are unique animals in that they are treated in veterinary clinics as patients but also used as experimental models in research studies. One of the most interesting features of canines is their intrabreed genetic homogeneity, resulting from the planned inbreeding performed to select certain visual and/or behavioral traits (Shearin and Ostrander, 2010). This remarkable genetic feature shared within breeds causes the very high occurrence of rare genetic diseases (e.g., the prevalence of dilated cardiomyopathy is 0.004% in human v.s. 58% in Doberman Pinscher), and thus it motivates researchers to use the canine as a model for studying common rare genetic diseases commonly found in both humans and canines (Hershberger et al., 2013; Hytonen and Lohi, 2016; Wess et al., 2017).

The advent of iPSCs led to the generation of several canine iPSC (ciPSCs) lines from various groups, including our own (Kimura et al., 2021; Nishimura et al., 2013; Tsukamoto et al., 2020, 2024; Yoshimatsu et al., 2021). Although current ciPSCs meet the validation criteria for pluripotency, their differentiation toward functional cells is yet to be achieved, delaying the application of ciPSCs in a variety of fields. This suggests that either the pluripotency of ciPSCs is insufficient to produce the functional cells or unknown factors are affecting their differentiation. Previous studies have shown heterogeneity of the different ciPSC lines cultured in the current medium, with reports of inconsistent pluripotency gene expression levels and differentiation potency *in vivo* (Kimura et al., 2021; Menon et al., 2021; Tsukamoto et al., 2024). Therefore, optimized culture conditions allowing maintenance of ciPSCs as a homogenous population may improve their differentiation potency.

Here, we have established NANOG-reporter ciPSC lines and used them to identify fibroblast growth factor (FGF), activin/TGF-β, and WNT signals as being critical for stabilizing the pluripotency of ciPSCs. Manipulating these signaling pathways increased the expression of NANOG in ciPSCs and suppressed the emergence of differentiated cells in ciPSC cultures after recovering them from frozen stocks. This conditioned medium presented robust differentiation potential of ciPSCs and stabilized ciPSCs regardless of the cell line or basal medium. Furthermore, these ciPSCs successfully differentiated into cardiomyocytes, displaying robust spontaneous rhythmic contraction and aligned sarcomere structure. We believe this robust medium condition would facilitate the utilization of ciPSCs in both clinical and basic research.

## Results

### Generation of NANOG-reporter ciPSC lines using CRISPR-Cas9 technology

**Figure 1:**
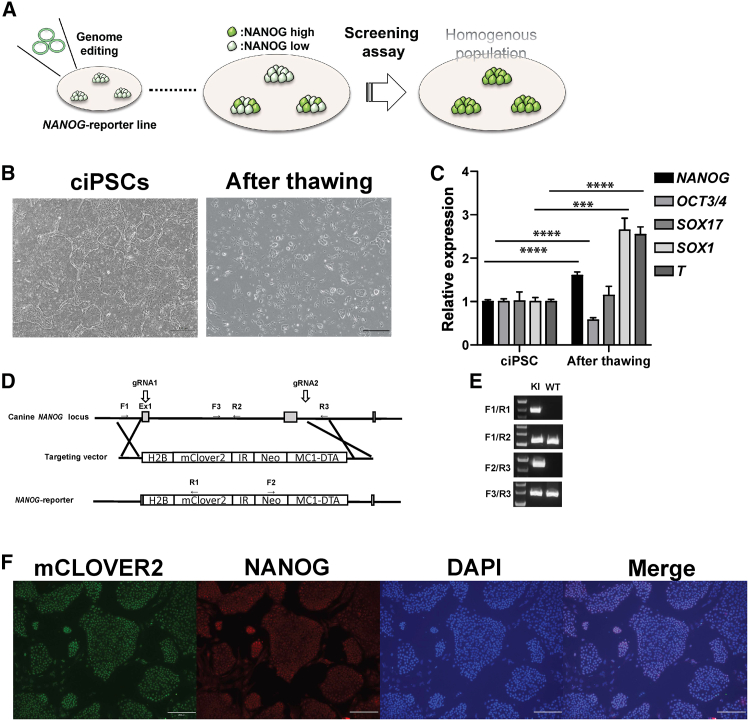
Establishment of the ciPSC NANOG-reporter line (A) Schematics showing NANOG-reporter strategy to define the culture condition for ciPSCs. (B) The representative image of ciPSCs, in culture (left) and after thawing (right). Scale bar, 500 μm. (C) Quantitative RT-PCR results of the representative genes in ciPSCs in culture (left) and after thawing (right) (mean ± SD of five replicates, independent experiments). ^∗∗∗^*p* ≦ 0.001, ^∗∗∗∗^*p* ≦ 0.0001. (D) Targeting strategy for knocking in the H2B-Mclover2 construct under the canine *NANOG* promoter locus. (E) Representative images of genotyping for the knocked-in construct in established reporter ciPSC lines. F1/R1: targeting between endogenous and knocked-in mClover2 sequence, F1/R2: left endogenous sequence, F2/R3: between knocked-in *Neo* resistant and endogenous sequence, and F3/R3: right endogenous sequence. (F) Representative immunofluorescence (IF) images of NANOG and mClOVER2 showing co-localized expression in ciPSCs. Scale bar, 200 μm.

We hypothesized that the pluripotent state of ciPSCs in the current medium was unstable, which resulted in variability in culture, gene expression, and differentiation (Figure 1A). This variability was well observed in the ciPSCs just recovered from the frozen stock, as some of these ciPSCs displayed fibroblast-like morphology in contrast to that of typical ciPSCs (Figure 1B). Quantitative RT-PCR of these ciPSCs revealed that the expression of *SOX1* and *T*, ectodermal and mesodermal markers in early embryo development, were approximately 3-fold higher in thawed ciPSCs compared to those maintained in culture. This suggested that ciPSCs tended to differentiate after thawing (Figure 1C). This spontaneous differentiation might be emphasized by the addition of Rock inhibitor into the medium to prevent ciPSCs from apoptosis, since the Rock inhibitor primes human iPSCs to differentiate toward the mesoendodermal lineage (Maldonado et al., 2016). We confirmed that the ciPSCs that were cultured long term with Rock inhibitor showed differentiation tendency through both morphology and gene expression levels (Figures S1A and S1B).

*NANOG* is a key marker gene for PSCs and has a critical role in the late phase of reprogramming where partially reprogrammed cells de-differentiate (Silva et al., 2009; Yu et al., 2007). Additionally, the expression of *NANOG* in ciPSCs greatly varies among cell lines (Tsukamoto et al., 2024). Given these facts, we decided to generate NANOG-reporter cell lines for monitoring the pluripotent state of ciPSCs. CRISPR-Cas9 based knockin strategy was employed to generate NANOG-reporter ciPSC lines as shown in Figure 1D. The knockin of reporter sequences in the endogenous *NANOG* locus was validated by PCR product size and Sanger sequencing (Figure 1E). The intact expression of *NANOG* in reporter cell lines was validated by reverse-transcription PCR (RT-PCR) (Figure S1C). The immunofluorescence staining confirmed that almost all ciPSCs expressed NANOG, and the expression of knockedin fluorescent protein, mCLOVER2, co-localized with NANOG in the nucleus (Figure 1F). Three of the seven reporter cell lines we obtained showed the highest level of reporter fluorescence and were used for subsequent experiments (Table S1).

### Screening assay for ciPSCs using NANOG-reporter cell lines

**Figure 2:**
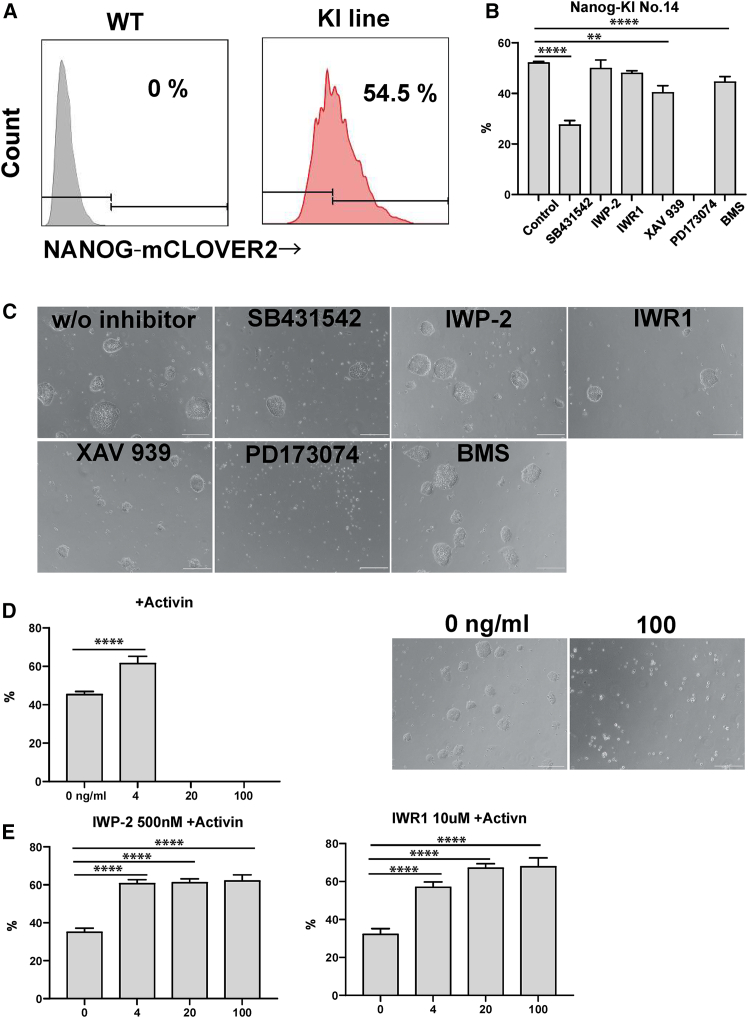
Inhibitor screening for optimizing the ciPSC culture condition (A) Representative FACS pattern of NANOG-mCLOVER2 expression in wild-type (left) and ciPSC reporter line (right) cultured in the control ciPSC medium. (B) The frequency of ciPSC_high_ cells in the NANOG-reporter ciPSC line (No.14) cultured with inhibitors (mean ± SD of three replicates, independent experiments). ^∗∗^*p* ≦ 0.01, ^∗∗∗∗^*p* ≦ 0.0001. (C) The representative images of ciPSCs cultured with inhibitors. Scale bar, 200 μm. (D) The frequency of ciPSC_high_ cells in the NANOG-reporter ciPSC line cultured with activin at different concentrations (mean ± SD of six replicates, independent experiments). The representative images of ciPSCs cultured with activin; scale bar, 500 μm. ^∗∗∗∗^*p* ≦ 0.0001. (E) The frequency of ciPSC_high_ cells in the NANOG-reporter ciPSC line cultured with 20 ng/mL activin + 500 nM IWP-2 or 10 μM IWR1 (mean ± SD of six replicates, independent experiments). ^∗∗∗∗^*p* ≦ 0.0001.

We utilized NANOG-reporter cell lines to investigate which signal cascade is critical for stabilizing the pluripotent state of ciPSCs by supplementing six inhibitors (activin/TGF-β [SB421542], WNT [IWP-2, IWR1, and XAV939], FGF [PD173074], and retinoic acid [BMS] inhibitors) in the ciPSC medium. We subsequently measured NANOG expression levels by flow cytometry (FCM) and set a stringent threshold above which positive NANOG expression was observed. The population above the threshold was termed ciPSC_high_ (Figure 2A). The number of ciPSC_high_ cells significantly decreased when SB431542, XAV939 or BMS were supplemented in the medium, although the morphology did not differ significantly from the control (Figures 2B and 2C). Interestingly, all the cells immediately disappeared when PD173074 was supplemented in the medium (Figures 2B and 2C). This result was consistent with previous reports demonstrating the essential role of FGF signal in ciPSC cultures (Goncalves et al., 2017; Nishimura et al., 2013). The decrease in the ciPSC_high_ population was also observed in other two reporter lines when they were cultured with SB431542, IWP-2, XAV939, or BMS (Figure S2A). Indeed, immediate cell disappearance was observed in these two lines cultured in media containing PD173074 (Figure S2A). Among these inhibitors, activin/TGF-β and FGF inhibitors consistently decreased the number of ciPSC_high_ population. Therefore, we decided to explore if the activation of these signal cascades increases the number of cells in the ciPSC_high_ population. We have supplemented either activin or bFGF to activate the activin/TGF-β and FGF signal, respectively. The ciPSC_high_ population significantly increased in the medium supplemented with 4 ng/mL activin, though ciPSCs disappeared at the concentration above 4 ng/mL (Figure 2D). On the other hand, the number of cells in the ciPSC_high_ population did not increase when bFGF was supplemented in the control medium (Figure S2B). This is probably because the current ciPSC medium has already been supplemented with enough bFGF. The activation of the activin/TGF beta pathway in concert with WNT signaling directs human PSCs toward mesendodermal differentiation (Naujok et al., 2014). In addition, activin treatment at low cell density induces cell death in human iPSC differentiation to the endodermal lineage (Thompson and Takebe, 2020). We speculated that the WNT pathway in the ciPSCs was activated when cultured in the current ciPSCs medium, and, thus, additional high concentrations of activin would promote differentiation. As expected, ciPSCs were able to be maintained in media containing WNT inhibitors and high concentrations of activin (Figure 2E). In addition, the number of cells in the ciPSC_high_ population significantly increased when cultured in the medium containing WNT inhibitors (Figure 2E). Since the number of cells in the ciPSC_high_ population was greater in the medium containing IWR1 compared to IWP-2, we further investigated the optimal concentration of IWR1. We quantified the number of cells in the ciPSC_high_ population to find a strong positive correlation with the increasing concentration of IWR1 to 50 μM, where almost all cells were found dead (Figure S2C). Thus, we have determined that 10 μM of IWR1 leads to the most effective expression of NANOG in the ciPSCs. The inhibition of the WNT pathway with both IWR1 and IWP-2 did not increase the number of cells in the ciPSC_high_ population (Figure S2D). Altogether, we have revealed that, while the FGF pathway is indispensable for ciPSC cultures, the activin/TGF beta pathway has a critical role in increasing the number of cells in the ciPSC_high_ population. In addition, we found that addition of WNT inhibitors stabilized the culture and prevented differentiation.

### Validating the effect of the AR medium on the pluripotency of ciPSCs

**Figure 3:**
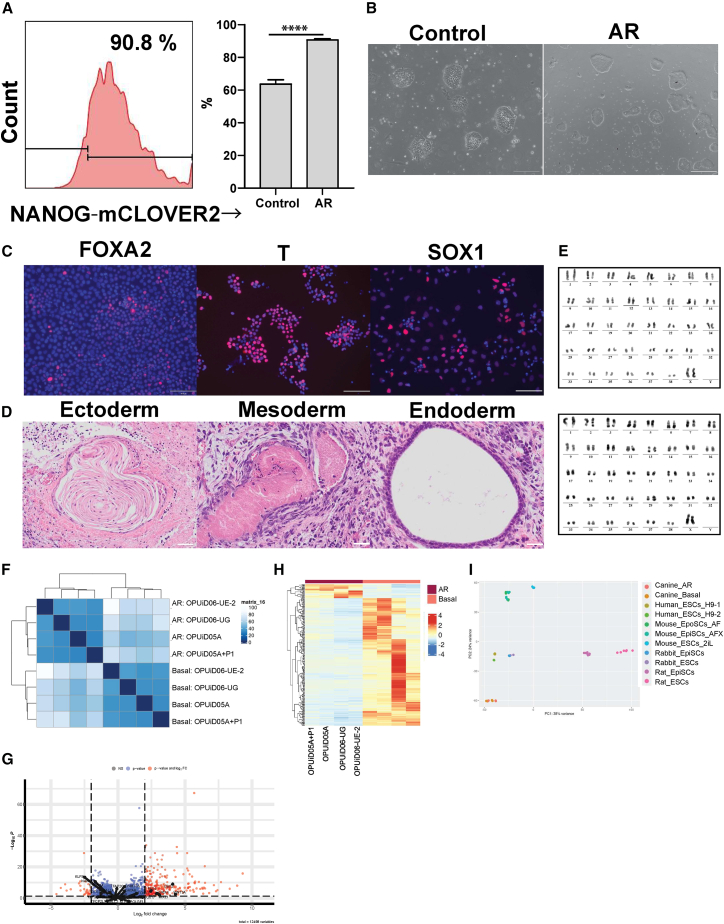
Validating the effect of AR medium on the pluripotent state of ciPSCs (A) Representative FACS pattern of NANOG-mCLOVER2 expression in ciPSC reporter line (no. 14) cultured with AR medium (right). The frequency of ciPSC_high_ cells in the NANOG-reporter ciPSC line cultured in control ciPSC or AR medium (left, mean ± SD of six replicates, independent experiments). ^∗∗∗∗^P ≦ 0.0001. (B) The representative images of ciPSCs in control ciPSC or AR medium. Scale bar, 200 μm. (C) Representative immunofluorescence (IF) images of FOXA2, T, and SOX1 in the cell differentiated from ciPSCs cultured with AR medium. Scale bar, 100 μm. (D) Representative images of hematoxylin eosin staining on the teratomas derived from ciPSCs cultured in AR medium. Scale bar, 500 μm. (E) Karyotyping analysis of ciPSCs cultured in AR medium. OPUiD05A (upper) and OPUiD06-UE-2 (lower). (F) Hierarchical clustering analysis for ciPSC lines cultured in either control ciPSC or AR medium. (G) Volcano plot showing differentially expressed genes between ciPSC lines cultured in control or AR medium. Pluripotent and lineage-associated genes are labeled. padj < 0.05; log2(fold change) > |2|. (H) Differential gene expression analysis for ciPSCs lines cultured in either control ciPSC or AR medium. (I) PCA analysis using the dataset of different species.

The ciPSC medium supplemented with activin and IWR1, hereafter called AR medium, significantly increased the number of cells in the ciPSC_high_ population compared to that in the control ciPSC medium (roughly 90% vs. 60%) (Figure 3A). Additionally, AR medium stably maintained ciPSCs and could be used to culture multiple ciPSC lines (Figure S3A). Results showed that the AR medium could be used to culture ciPSCs independent of the cell line or basal medium (Figure S3B). Although the AR medium maintained ciPSCs with high expression of NANOG, it was uncertain how this medium affected the pluripotency of ciPSCs. Hence, we investigated the cell morphology, differentiation potential, and karyotyped the ciPSCs cultured in AR medium. The ciPSCs cultured in AR presented slightly domed and uniform morphology in contrast to the typical ciPSCs, which were flattened with heterogeneous morphology (Figure 3B). When these cells were cultured in the optimized condition previously reported for the differentiation of rabbit PSCs, they immediately differentiated into different cell types *in vitro*, including SOX1-positive ectoderm, FOXA2-positive endoderm, and T-positive mesoderm cells (Figure 3C) (Kobayashi et al., 2021). The transplantation of these ciPSCs into immune deficient mice generated teratomas composed of the cells derived from all three germ layers (Figure 3D). This was confirmed for all three ciPSC lines, including OPUiD06-UG, which could not generate teratomas when cultured in the non-AR control medium. All transplanted mice formed teratomas containing all three germ layers (Figure S3C). The ciPSCs were routinely passaged as single cells and maintained normal karyotypes after more than 10 passages (Figure 3E). Spontaneous differentiation of ciPSCs is a common issue encountered when recovering ciPSC from frozen stock. Culturing thawed ciPSCs in AR medium decreased the emergence of the fibroblast-like differentiated cells. This observation was confirmed by qPCR, where the expression of differentiation markers, SOX17 and T, dramatically decreased in the cells recovered in AR medium compared to those in the control ciPSC medium (Figure S3D). Collectively, ciPSCs cultured in the AR medium were pluripotent and displayed a homogenous population compared to the cells cultured in control ciPSC medium. In addition, the AR medium did not induce chromosomal abnormality in the ciPSCs and improved the differentiation potency of ciPSCs *in vivo*. These results motivated us to suggest the AR medium as a more appropriate medium for ciPSC cultures than the control medium currently used.

### Gene expression profile of ciPSCs cultured in AR medium

Next, we examined the transcriptomes of multiple ciPSC lines cultured in either the AR or the control ciPSC medium. Hierarchical clustering and principal-component analysis (PCA) showed a clear separation between the ciPSCs cultured in AR and control medium (Figures 3F and S3E), with differential gene expression (DGE) analysis revealing a more homogeneous gene expression pattern of ciPSC lines cultured in AR medium (Figure 3H). The ciPSCs cultured in AR medium showed downregulation of lineage-specific genes (*WNT3A*, *MIXL1*, *PAX6*, *GATA4*, and *GATA6*) but no significant difference in the expression of pluripotency genes. This suggested that the AR medium likely suppressed the differentiation of ciPSCs rather than enhancing their pluripotency (Figure 3G). These gene expression patterns were consistent between cell lines (Figure S3F). Although almost all naive marker genes were expressed at similar levels among the samples, the expression of *GBX2* in ciPSCs was significantly downregulated in the AR medium, implying the species-specific molecular role of *GBX2* in ciPSCs (Figure 3G). Altogether, these results suggested that the AR medium altered the gene expression pattern of ciPSCs. Additionally, lineage marker genes were downregulated in the ciPSCs cultured in the AR medium and homogenized the global gene expression between cell lines. We further performed a cross-species comparison of these transcriptomic data with published human ESC lines (Chu et al., 2016; Luo et al., 2020), mouse EpiSCs and ESCs (Kinoshita et al., 2021a), rabbit EpiSCs and ESCs (Kobayashi et al., 2021), and rat EpiSCs and ESCs (Iwatsuki et al., 2023). Correlation matrix analysis and PCA exhibited a close correlation of human ESC and ciPSC lines, while ciPSCs were less correlated with other species PSCs (Figures 3I and S3G).

### ciPSCs cultured in AR medium produce cardiomyocyte cells coupled with functional structure

Although previous studies have characterized various ciPSC lines, their directed differentiation *in vitro* into specific cell types has remained elusive. This lack of understanding of differentiation potential has delayed the utilization of ciPSCs in a variety of fields. We have shown that the AR medium improves the differentiation potency of ciPSCs *in vitro* through teratoma formation. We next wanted to investigate whether the AR medium further enables ciPSCs to give rise to functional derivatives. Given that the canine is one of the most frequently used cardiovascular models for human medicine (Camacho et al., 2016), we attempted to differentiate ciPSCs to cardiomyocytes and investigate their characteristics.

**Figure 4:**
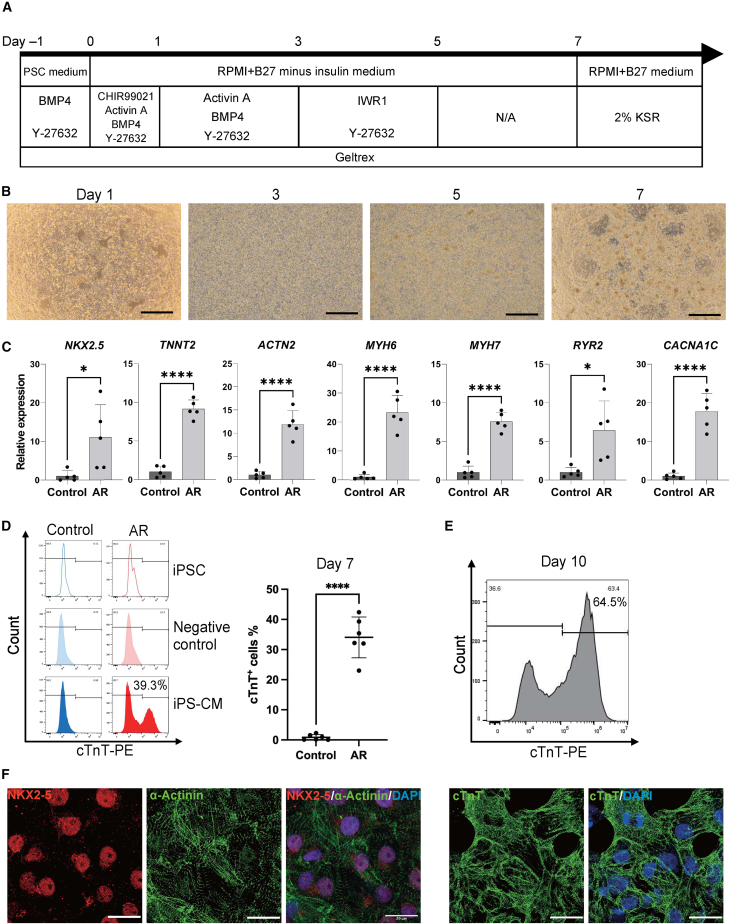
Cardiac differentiation from ciPSCs in AR and control medium (A) The scheme of cardiac differentiation protocol using ciPSCs. (B) The representative images of the differentiated cells from ciPSCs in AR medium at each time point. Scale bar, 500 μm. (C) Quantitative RT-PCR results of the representative gene expressions in differentiated cells on day 7 derived from ciPSCs cultured in control and AR medium (mean ± SD of five replicates, independent experiments). ^∗^*p* ≦ 0.05, ^∗∗∗∗^*p* ≦ 0.0001. (D) Representative histograms of cTnT expression in iPSCs and differentiated cells (iPS-CM) on day 7 derived from ciPSCs cultured in the control and AR medium (left). Negative control: unstained differentiated cells. The frequency of cTnT-positive cells (right, mean ± SD of six replicates, independent experiments). ^∗∗∗∗^*p* ≦ 0.0001. (E) Representative histogram image of cTnT expression in differentiated cells on day 10 from ciPSCs cultured in AR medium. (F) Representative IF images of NKX2-5, α-Actinin, and cTnT in the differentiated cells on day 12 derived from ciPSCs cultured in AR medium. Scale bar, 20 μm.

Canine iPSCs cultured in either control ciPSC or AR medium were differentiated to cardiomyocytes based on the previously established human protocols with the modification of differentiation timeline and the concentration of cytokines (Burridge et al., 2014; Lian et al., 2012) (Figure 4A). Both groups underwent significant morphological changes during differentiation (Figures 4B and S4A). Notably, ciPSCs in AR medium formed a more homogeneous population compared to those in the control medium, mirroring the effect of AR medium on ciPSCs homogeneity (Figure 4B). At around day 7 after differentiation, the cardiomyocytes derived from ciPSCs in AR medium (ciPSC-CM_AR_) began synchronized beating, a hallmark of functional cardiomyocytes (Video S1), whereas the control medium-derived cells did not. Quantitative RT -PCR of the differentiated cells on day 7 confirmed that key cardiomyocyte-associated genes, including *NKX2-5*, *TNNT2*, *ACTN2*, *MYH6*, *MYH7*, *RYR2*, and *CACNA1C*, were significantly upregulated in the ciPSC-CM_AR_ compared to the differentiated cells in the control group (Figure 4C). Conversely, mesodermal and cardiac progenitor genes, including PDGFRA and ISL1, were downregulated in those cells compared to the differentiated cells derived from control ciPSCs, whereas the expression levels of MESP1 and KDR showed no significant differences between the two groups. These results suggested that the ciPSC-CM_AR_ were in a developmentally more mature state than the control cells, which had only slightly differentiated into the cardiac lineage (Figure S4B). Of note, FCM analysis of day 7 differentiated cells showed that cardiac troponin T (cTnT), a crucial protein involved in calcium-mediated actin-myosin interactions (Sharma et al., 2004), was expressed in more than 30% of ciPSC-CM_AR_ (Figure 4D). On day 10, the frequency of cTnT-positive cells was greater than half of the differentiated cells (Figure 4E). The sarcomere is the highly ordered multiprotein complex responsible for the generation of active and passive forces of the heart (Crocini and Gotthardt, 2021). Immunostaining revealed that aligned sarcomere structures were formed in the ciPS-CM_AR_ on day 12, confirmed by the robust expression of NKX2-5, cTnT, and α-Actinin (Figure 4F). In contrast, the cells from the control group lacked these cardiomyocyte markers and failed to form sarcomeres (Figure S4C). Taken together, ciPSCs cultured in AR medium successfully differentiated into the cardiomyocytes, displaying synchronized contractions as well as sarcomere alignment. In contrast, the ciPSCs in the control medium failed to produce those derivatives, underscoring the critical role of AR medium in cardiomyocyte differentiation.

Video S1. The synchronized contraction of ciPSC-CMAR at differentiation day 10

## Discussion

In this study, we identified an optimal universal culture medium (AR) that could maintain ciPSCs in the undifferentiated state regardless of the cell line or basal medium used. The ciPSC lines cultured in AR medium exhibited distinct and homogenous gene expression patterns compared to the ciPSCs in the control ciPSC medium. They also displayed improved differentiation potency *in vivo* (teratoma formation) and *in vitro* (the induction of cardiomyocytes). The canine is an attractive and unique animal model sharing several features with humans, including often a shared living environment and lifestyle (Hoffman et al., 2018). Thus, defined culture conditions allowing ciPSCs to differentiate into functional cells can be valuable for developing canine disease models with potential wider applications to translational medicine.

CRISPR-Cas9 technology has been widely used for generating knockin PSC lines derived from different animal species, though it has been unclear if the same technology can be applied to ciPSCs (Iwatsuki et al., 2023; Kobayashi et al., 2021; Shishido et al., 2018). In this study, we generated NANOG-reporter ciPSC lines with CRISPR-Cas9 technology and utilized it to validate the critical signal pathways involved in ciPSC maintenance in culture. We confirmed that the designed plasmid construct was successfully introduced into the targeted locus of the canine *NANOG* gene and properly expressed the fluorescent proteins. The reporter strategy is often employed to trace and understand the trajectory of developing cells showing dynamic lineage specification. Therefore, the NANOG reporter system along with the robust *in vitro* culture provides a powerful tool to elucidate canine development.

The ciPSCs present controversial characteristics (e.g., the expression of SSEA4) that are not consistent with the cell lines or reports of other species. Hence, the state of ciPSCs remains under discussion (Kimura et al., 2021; Menon et al., 2021; Tsukamoto et al., 2024). We revealed that ciPSCs stabilized their pluripotency in media containing activin, WNT inhibitor, and bFGF, the components that are generally used for culturing EpiSCs of other species (Kinoshita et al., 2021b). In addition, the global gene expression pattern of ciPSCs in AR medium was more similar to that of EpiSCs (mouse, rat, and rabbit) than ESCs. Given these facts, ciPSCs cultured in AR medium would be categorized as the EpiSCs state, also known as primed state.

We also found that ciPSCs easily differentiated into fibroblast-like mesodermal cells after freezing and thawing. The AR medium suppressed the emergence of these unfavorable cells and decreased the expression of lineage-specific markers. These results suggested that some of the ciPSCs cultured in the previous medium were “ready to differentiate,” eventually forming a heterogeneous population that impaired the pluripotency of the ciPSCs. We confirmed this hypothesis by using a ciPSC line that cannot form a teratoma despite being able to be maintained and express pluripotency markers in culture. We showed that teratomas could be formed from this cell line when cultured in the AR medium. Additionally, ciPSCs in the AR medium could differentiate into cardiomyocytes that displayed synchronized contraction, one of the crucial functions of the heart. This could not be observed in the derivatives of ciPSCs cultured in the control ciPSC medium. Consequently, we concluded that the AR medium improved the developmental potential of ciPSCs, allowing them to give rise to functional derivatives.

In this study, we utilized five different ciPSC lines derived from either the peripheral blood or urine-derived cells of the five different donors. In addition, the AR medium has been used across three different laboratories in two countries, yielding reproducible results. These findings suggest that the AR medium can support ciPSC culture regardless of the original cell source or lab environment, though further validation will be necessary. It is important to note that these ciPSC lines were adapted to the AR medium at early passages (passage 8–11). In future studies, it will be informative to examine whether ciPSCs with different characteristics (e.g., late passage, developed from patient samples, etc.), can be adapted to and maintained in the AR medium. During the course of this study, we occasionally observed slowed cell proliferation. This was largely resolved by reducing the concentration of IWR1. Optimizing the concentration of WNT inhibitor will be a key step in adapting ciPSCs to the AR medium.

Overall, our study proposed a stable ciPSC culture system with potential applications to both canine and human disease treatment models.

## Methods

### Animals

Four- to 6-week-old non-obese diabetic (NOD)/severe combined immunodeficiency (SCID) male mice were purchased from Charles River Laboratories Japan, Inc. (Kanagawa, Japan), and used in this study. All experiments were performed in accordance with the animal care and use committee guidelines of Osaka Metropolitan University.

### Maintenance of ciPSCs

Four female ciPSC lines (OPUiD05A, OPUiD04B, OPUiD06-UE-2, and OPUiD01-UB-1) were derived in previous literatures (Kimura et al., 2021; Tsukamoto et al., 2024). One female ciPSC line (OPUiD06-UG) was established in-house according to the previous report (Tsukamoto et al., 2024). Information for each cell line can be found in Table S1. Canine iPSCs were maintained on iMatrix-511 silk (Nippi, Tokyo, Japan) in StemFit AK02N (Ajinomoto, Tokyo, Japan) or StemFit supplemented with 5–10 μM IWR1 (Nacalai Tesque, Kyoto, Japan) and 20 ng/mL activin A (R&D Systems, Minnneapolis, MN). Canine iPSCs were also maintained on Vitronectin (Thermo Fisher Scientific, Waltman, MA) in StemFlex (Thermo Fisher Scientific) or StemFlex supplemented with 10 μM IWR1 and 20 ng/mL activin A. For passaging, confluent ciPSCs colonies were dissociated with TrypLE Express (Thermo Fisher Scientific) or 0.5 mM EDTA/D-PBS and reseeded onto the plate containing ciPSC medium with 10 μM Y-27632 (Fujifilm, Osaka, Japan), pre-coated with extracellular matrix. The cells were routinely passaged every 4 days.

### Vector construction and gene introduction

To construct knockin vectors targeting the *NANOG* locus, 5′ and 3′ homology arms amplified from genomic DNA of ciPSCs, dClover2-C1 (addgene: 54577), H2B-IRES-Neo amplified from pH2B_mCherry_IRES_*neo*3 (addgene: 21044), and MC1-promoter-driven diphtheria toxin A (DTA) cassette amplified from pDEST-R4R3-MC1DTA (addgene: 139521) were assembled with NEBuilder HiFi DNA assembly master mix (New England Biolabs, Ipswich, MA). For efficient gene editing, the clustered regularly interspaced short palindromic repeats (CRISPR)-associated protein 9 (CRISPR-Cas9) and single guide RNAs (sgRNAs) were co-transfected with knockin vectors. The sgRNAs targeting the sequence of *NANOG* (sgRNA1: 5′-gctcctccccaatgcccgcc-3′, sgRNA2: 5′-ggtcgtggtgcccaggtcct-3′) and Alt-R S.p. HiFi Cas9 Nuclease V3 (IDT, Coralville, IA) were used for gene editing.

Reverse transfection was performed using Lipofectamine 3000 (Thermo Fisher Scientific) according to the manufacturer’s instructions. Briefly, 100 μL of Opti-MEM (Thermo Fisher Scientific) containing 50 ng/μL Cas9 protein, 50 ng/μL sgRNAs, lipofectamine complex, and knockin vector were incubated for 15 min at room temperature. After incubation, the mixed solution was added into 60%–80% confluent ciPSCs cultured in a 12-well plate. Forty-eight hours later, 200 μg/mL G418 (Sigma-Aldrich, Burlington, MA) was added to the culture medium for selection. After the selection, G418-resistant ciPSCs colonies were cloned and validated for having correct insertion of knockin construct by PCR using primers in Table S3.

### *In vitro* differentiation of ciPSCs into three germ layers

The differentiation protocol used here was designed based on the previous reports (Kobayashi et al., 2017, 2021). All the differentiation were performed using basal medium, aFB27 medium, which is composed of L (Thermo Fisher Scientific) supplemented with 1% B27 supplement (Thermo Fisher Scientific), 0.1 mM NEAA, 100 U/mL penicillin-0.1 mg/mL streptomycin, 0.1% polyvinyl alcohol, and 2 mM L-glutamine. For mesendoderm induction, dissociated ciPSCs were seeded on a vitronectin-coated dish at 200,000 cells per well in 12-well plates and cultured in a mesendoderm induction medium for 24–48 h. The mesendoderm induction medium contained an aFB27 medium supplemented with 100 ng/mL activin A, 3 μM CHIR99021 (R&D Systems), and 10 μM Y-27632. For definitive endoderm induction, mesendoderm induction medium was replaced with definitive endoderm induction medium after washing with PBS once, and cells were cultured for 3 more days. Definitive endoderm induction medium was composed of modified aFB27 medium supplemented with 100 ng/mL activin A and 0.5 μM BMPi (LDN193189: Selleck, Houston, TX). For neuroectoderm (NE) induction, dissociated ciPSCs were seeded on a vitronectin-coated dish at 200,000 cells/well in 12-well plates and cultured in NE induction medium for 72 h. NE induction medium was composed of modified aFB27 medium supplemental with 10 μM TGF-βi (SB43152, Nacalai Tasque), 0.5 μM BMPi, and 10 μM Y-27632.

### Teratoma formation

Teratoma formation from ciPSCs was conducted as described previously (Tsukamoto et al., 2024). Briefly, approximately 1 × 10e6 ciPSCs were transplanted into either the subcutaneous tissue or testes of male NOD/SCID mice. Two to 3 months later, the teratomas were dissected and analyzed by hematoxylin and eosin staining.

### Small-molecule screening using NANOG-reporter ciPSC lines

NANOG-reporter ciPSC lines (no.10, 14, and 15) were used for screening small molecules in the ciPSC culture. The reporter lines were passaged at 5,000 cells per well in 24-well plates and cultured in StemFit supplemented with 10 μM TGF-β inhibitor (SB431542), WNT inhibitors (100 nM IWP-2, 10 nM IWR1, and 10 μM XAV939, all from Nacalai Tesque), FGF receptor inhibitor (PD173074: Selleck), and retinoic acid receptor) inhibitor (BMS: MedChemExpress, Monmouth Junction, NJ) for 4 days. After 4 days of culture, ciPSCs in each condition were analyzed by microscope and FCM targeting the frequency of mClover2 positive cells.

### Differentiation of ciPSCs into cardiomyocyte cells

Canine iPSCs cultured in either control ciPSC or AR medium were passaged onto Geltrex (Thermo Fisher Scientific) and cultured in StemFlex or AR medium with 10 μM Y-27632 and 20 ng/mL BMP4 (R&D Systems). After 24 h, ciPSCs were further cultured in the differentiation medium supplemented with 6 μM CHIR99021, 20 ng/mL activin A, 20 ng/mL BMP4, and 10 μM Y-27632 (day 0). After 24 h culture, CHIR99021 was removed (day 1). On day 3, the differentiated cells were cultured in the RPMI+B27 minus insulin medium supplemented with 5 μM IWR1 and 10 μM Y-27632 for 48 h and subsequently cultured in the medium without any supplement for additional 48 h (day 5–7). On day 7, the medium was changed to the RPMI+B27 medium supplemented with 2% knockout serum replacement (Thermo Fisher Scientific). The differentiated cells were further cultured until analysis. The RPMI+B27 minus insulin medium was composed of RPMI1640, 1 × B27 minus insulin (Thermo Fisher Scientific), 0.1 mM NEAA, 100 U/mL penicillin-0.1 mg/mL streptomycin, sodium pyruvate, and 100 μg/mL L-ascorbic acid. In the composition of the RPMI+B27 medium, the 1× B27 minus insulin supplement was replaced with the 1× B27 supplement (Thermo Fisher Scientific), compared to the RPMI+B27 minus insulin medium.

### Flow cytometry analysis

Canine iPSCs were analyzed by the SH800 cell sorter (SONY, Tokyo, Japan) or BD Canto II (BD Biosciences, Franklin Lakes, NJ). cTnT staining was conducted as described previously (Waas et al., 2019). In brief, the collected cells were fixed with 2% formaldehyde (w/v) in D-PBS for 20 min and permeabilized with 0.5% saponin (w/v)/0.5% BSA (w/v)/D-PBS for 15 min at room temperature. The samples were incubated with PE-conjugated mouse anti-cTnT antibody (BD Biosciences; clone 13–11) for 45 min at room temperature. Data were acquired on NovoCyte Flow Cytometers (Agilent, Santa Clara, CA) and were analyzed using FlowJo v.10 (FlowJo).

### Quantification and statistical analysis

#### Flow cytometry analysis

Fluorescence-activated cell sorting (FACS) data were analyzed and visualized by FlowJo software (BD Biosciences).

#### Bioinformatic analysis

For ciPSC bulk RNA sequencing (RNA-seq) analysis, single-read sequencing reads were screened for quality using fastQC (v.0.11.9) and then aligned to the Ensembl ROS Cfam 1.0 dog genome using STAR (v.2.7.8a) (Dobin et al., 2013). Raw gene counts were generated using subread featureCounts (v.2.0.0) (Liao et al., 2014) and passed into the DESeq2 workflow (Love et al., 2014) for subsequent DGE analysis. Data were normalized and regularized log transformed prior to performing PCA. Contrasts were created to perform Wald testing on shrunken log2 fold changes (type = apeglm) (Zhu et al., 2019) and used for data visualization of significant differentially expressed genes (padj < 0.05, log2FoldChange > |2|). This included generating volcano plots using EnhancedVolcano and heatmaps using pheatmap (v.1.0.12). (Blighe et al., 2024; Kolde, 2018)

The following raw datasets were downloaded for cross-species analysis: human ESCs (GSE75748 [Chu et al., 2016], GSE174070 [Consortium, 2012]), mouse EpiSCs and ESCs [GSE131556; Kinoshita et al., 2021a], rabbit EpiSCs and ESCs [E-MTAB-10892; Kobayashi et al., 2021], and rat EpiSCs and ESCs [GSE220805; Iwatsuki et al., 2023]). Paired sequencing reads were aligned to its own reference genome (human hg38, mouse GRCm39, rabbit OryCun2.0, and rat rn7) to generate raw gene counts. Ensembl gene stable IDs for each species were converted to orthologous mouse gene stable IDs using biomaRt (Durinck et al., 2005, 2009). Only orthologous genes that were identified in all five species were used for downstream analysis. The gene length of each orthologous gene in their respective species was calculated and used as a normalization factor. Subsequent analysis was performed as described earlier. Correlation coefficients were calculated using normalized counts and visualized using ConsensusClusterPlus (Wilkerson and Hayes, 2010).

#### Statistical analysis

Quantitative data were generated with Microsoft Office Excel or GraphPad Prism and presented as mean ± SD. All statistical details of the experiments can be found in the figure legends.

## Resource availability

### Lead contact

Further information and requests for resources and reagents should be directed to the lead contact, Toshiya Nishimura (tnishimu.kbb@osaka-u.ac.jp).

### Materials availability

Unique reagents generated in this study are available from the lead contact with a materials transfer agreement.

### Data and code availability

• The Gene Expression Omnibus (GEO) accession number for the Bulk RNA-seq data generated in this study is GSE289292. All other publicly available datasets used in this manuscript are listed in Tables S1 and S2. No new code was generated in this study.
• All code will be deposited in GitHub and available for public access on the date of manuscript publication.

## Acknowledgments

We thank the members of Takebe lab, particularly Naomi Maeda for secretarial support, and the members of Hirabayashi lab. This study was supported by the Center for Medical Research and Education, Graduate School of Medicine, The University of 10.13039/501100004206Osaka. We acknowledge the NGS core facility at the Research Institute for Microbial Diseases of The University of Osaka for the sequencing. This work was supported by grants from 10.13039/501100001691JSPS KAKENHI (grant numbers 21H02378 and 24K21916) and a grant from the 10.13039/100016512Center for Companion Animal Health (CCAH) at UC Davis (grant number 2023-60-F).

## Author contributions

T.N. designed the study, performed experiments, analyzed data, and wrote the manuscript. K.K. performed the differentiation of ciPSCs and with A.K. analyzed data and edited the manuscript. A.B. analyzed the gene expression of ciPSC-derived cardiomyocytes. K.J.I. performed bioinformatics analysis and edited the manuscript. K.S. performed teratoma formation experiments and analyzed the data. H.S. performed quantitative reverse-transcription PCR experiments in cardiac differentiation. M.T. performed karyotyping experiments and analyzed the data. C.F. performed molecular characteristics of reporter ciPSC lines. M.H. and S.H. supervised the projects and shared materials.

## Declaration of interests

T.N., K.K., and S.H. have a patent application related to this work.

## Supplemental information

Document S1. Figures S1–S4, Tables S1–S3, and supplemental methods

Document S2. Article plus supplemental information
