# Supplementary material for: Signaling pathway-based culture condition improves differentiation potential of canine induced pluripotent stem cells
Source: Stem Cell Reports. 2025 Sep 18;20(10):102640. doi: 10.1016/j.stemcr.2025.102640 (PMC12790732; doi:10.1016/j.stemcr.2025.102640)
Supplement: Document S1. Figures S1–S4, Tables S1–S3, and supplemental methods [file mmc1.pdf]

Stem Cell Reports, Volume *20*

Supplemental Information

Signaling pathway-based culture condition improves differentiation potential of canine induced pluripotent stem cells

Toshiya Nishimura, Kazuto Kimura, Kyomi J. Igarashi, Kohei Shishida, Hiroko Sugisaki, Masaya Tsukamoto, Aadhavan Balakumar, Chihiro Funamoto, Masumi Hirabayashi, Amir Kol, and Shingo Hatoya

Cell line	Sub clone	Figure number	iPSC derivation vector	Original cell	Feeder cell at derivation	Reference	Initial passage number
OPUiD05A		1, S1, 2A (WT), 3C-I, S3D-G	Sendei virus vector containing human OCT3/4, KLF4, SOX2 and C-MYC	Peripheral blood mononuclear cells (PBMCs)	+	Kimura et al., 2021	8
	KI-No.10	S2A					
	KI-No.14	2, S2B-D, 3A-B					
	KI-No.15	S2A					
OPUiD01-UB-1		S3A	Sendai virus containing canine OCT3/4, KLF4, SOX2, C-MYC, LIN28, and NANOG	Urine-derived cells (UCs)	+	Tsukamoto et al., 2024	8
OPUiD06-UG		3F-I, S3A, S3C, S3E-G, 4,S4	same as OPUiD01-UB-1	UCs	+		11
OPUiD04B		S3A	same as OPUD05A	PBMCs	+	Kimura et al., 2021	8
OPUiD06-UE-2		3E-I, S3A, S3C, S3E-G	same as OPUiD01-UB-1	UCs	—	Tsukamoto et al., 2024	11

**NANOG-reporter lines**	**No. 6**	**No. 8**	**No. 10**	**No. 11**	**No. 13**	**No. 14**	**No. 15**
KI locus determination	○	○	○	○	○	○	○
NANOG expression	○	○	○	○	○	○	○
NANOG-mCLOVER2 (%)	2.4	0.32	27.3	0.78	0.086	49.7	37.6

Table S1: The details about each cell line used in this study, related to Figure 1-4

	Name	Sequence 5' - 3'	
Knock-in	F1	aaggaacctgtcacaaacgctttg	
	R1	tacttggtgatggccttagtaccct	
	R2	gccaaaccaactactagtgtcgt	
	R3	catgcccctagttggttaaaggtac	
	F2	caggatgatctggacgaagagca	
	F3	tcattcccacctgagtagccaca	
qRT-PCR		Sequence 5' - 3'	
		Forward	Reverse
Early embryo development	NANOG	cggatccagctcctccccaa	aggaaggaagaggagagacggt
	OCT 3/4	aaccccgaggagtcccaaga	gcagagcctcaaaacggcag
	T	gggtactcccaatggggttgg	tagggggatggacggtggtt
	SOX17	cactttgtgtgcaagcccga	agtagtacacggccgagctg
	FGF5	ggaaaactccatgcaagtgcca	cttagccttgccccgcttgt
	SOX1	tggaaggtcatgtccgaggc	tcttgagcagcgtcttggtct
	GAPDH	ccctgagctgaacgggaagc	ttgaggggtccctccgatgc
Cardio differentiation	MYH7	acctgtcgagaagggcaaag	gggttgtctatcacccctgg
	MYH6	gcagtaaaggcaaaggaggc	agcttattcagattctcccggtg
	TNNT2	aggagtccaaaccaaagccc	tccggtggatgtcgtcaaag
	ACTN2	acgtgggcttatggcaaaga	ctctcgaaggcctcgtgttt
	RYR2	cccaacgcagcaaggaaaaa	tttgctggcactgatggtct
	CACNA1C	ctgacccgtgagcacacat	ctcccatagttggaacctccag
	MESP1	gcccgtcgtctccgctctttcc	cgatgctcacagacagggctcca
	KDR	cgagacacttgtggaagactca	tcctgggcaccttctactatga
	PDGFRA	atcttttcccttggcggcaca	cttcactggtggcgtggtca
	ISL1	gatttccctatgtgttggttgcg	gcatttgatcccgtacaacctgata
	NKX2.5	caagtgtgcgcctgcattt	gcagtgagcacagctctttc
	GAPDH	catgtttgtgatgggcgtgaacca	tttggctagaggagccaagcagtt

Table S2: Primer list used in this study, related to Figure 1 and 4

Primary Antibody	Source	Identifier	Dilution
Anti-GFP	Abcam	AB13970-100	1000
Anti-NANOG	Novus biologicals	NOV-NB100-58842-0.1	250
Anti-FOXA2	Abcam	ab108422	1000
Anti-T	Abcam	ab209665	1000
Anti-SOX1	R&D	AF3369	50
Anti-NKX2-5	Abcam	ab97355	50
Anti-α-Actinin (Sarcomeric)	Sigma-Aldrich	A7811-100UL	400
Anti-cTnT	Thermo Fisher Scientific	MA5-12960	200
**Secondary Antibody**	**Source**	**Identifier**	**Dilution**
Anti-chicken Alexa488	Thermo Fisher Scientific	A78948	1000
Anti-rabbit Alexa 594	Thermo Fisher Scientific	A3275	1000
Anti-goat Alexa 594	Thermo Fisher Scientific	A-11058	1000
Anti-mouse Alexa488	Thermo Fisher Scientific	A21202	1000
Anti-rabbit Alexa 594	Thermo Fisher Scientific	A21207	1000

Table S3: Antibody list used in this study, related to Figure 1, 3, and 4

SUPPLEMENTAL METHODS

Quantitative reverse transcription PCR

Total RNA was extracted using NucleoSpin® RNA Plus (MACHEREY-NAGE: Nordrhein-Westfalen, Germany) and cDNA was synthesized using ReverTra Ace® qPCR RT Master Mix (TOYOBO: Osaka, Japan) according to the manufacturer's instructions. qRT-PCR was performed using PowerTrack™ SYBR Green Master Mix for qPCR (Thermo Fisher Scientific) and analyzed by the QuantStudio 3 or StepOnePlus real-time PCR system (Thermo Fisher Scientific) using the comparative CT method. The primers sequences used in the paper are listed in **Table S2**.

Preparation of RNA-sequencing libraries

Total RNA was extracted using NucleoSpin® RNA Plus (MACHEREY-NAGE: Nordrhein-Westfalen, Germany). Library preparation was performed using a TruSeq stranded mRNA Library Prep kit (Illumina: Research PI, SD) according to the manufacturer's instructions. Sequencing was performed on NovaSeq 6000 (Illumina) in 101-base single-read mode. Subsequent bioinformatic analysis is detailed below.

Immunofluorescence analysis

Samples were fixed with 4% paraformaldehyde (w/v) for 10 min and permeabilized with 0.1 % Triton X-100 (w/v) for 10 min at room temperature. After incubating with blocking buffer (MAXblock™ Blocking Medium: Active Motif, Carlsbad, CA) for 30 min, the samples were incubated with primary antibodies at 4 °C overnight. Next, the samples were incubated with fluorescent-conjugated secondary antibodies for 1 h. followed by mounting using VECTASHIELD Vibrance Antifade Mounting Medium with DAPI (Vector Laboratories, Newark, CA). The antibodies used are listed on **Table S3**. Specimens were observed and analyzed using EVOS M5000 (Thermo Fisher Scientific) or a Leica TCS SP8 STED 3X confocal laser scanning microscope (Leica Microsystems, Wetzlar, Germany).

Karyotyping analysis

ciPSCs were incubated with 0.05 mg/mL colcemid (Thermo Fisher Scientific), trypsinized, and incubated with 0.075 M KCl. The cells were then fixed in acetic acid:methanol (1:3), stained with quinacrine mustard and Hoechst 33258, and observed using confocal laser microscopy (LSM980; Carl Zeiss, Oberkochen, Germany).

Supplemental figure 1

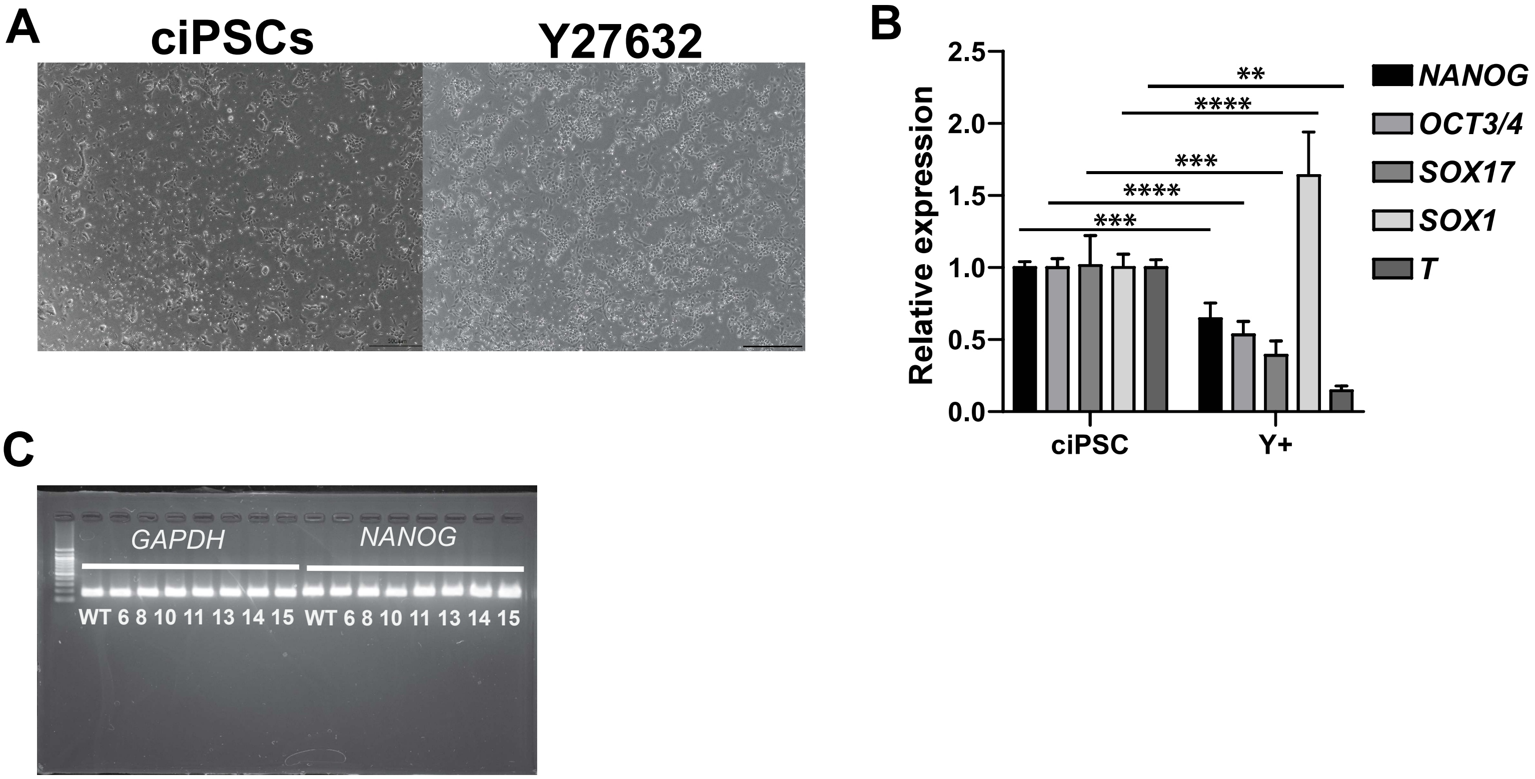

Supplemental figure 2

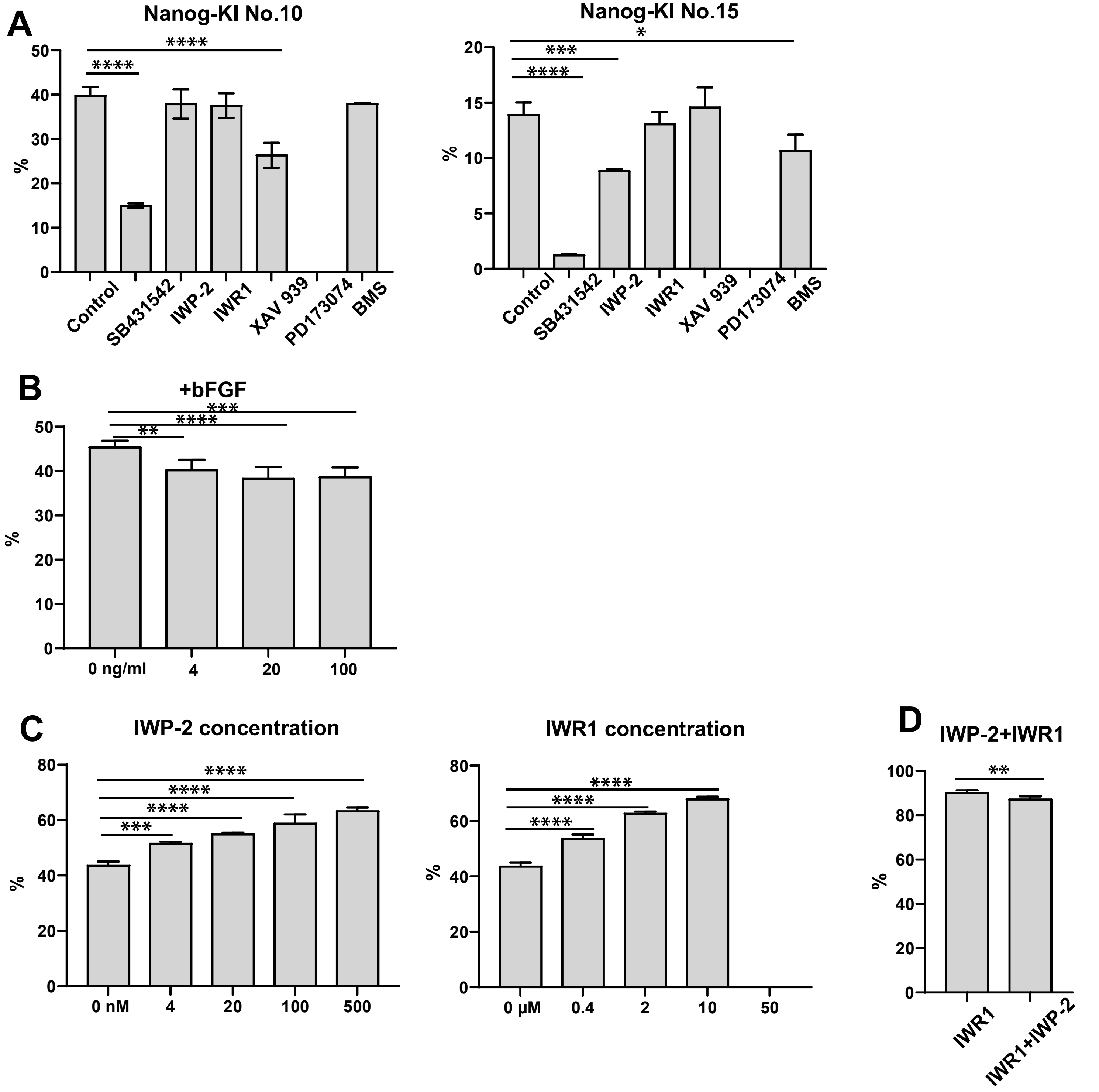

Supplemental figure 3

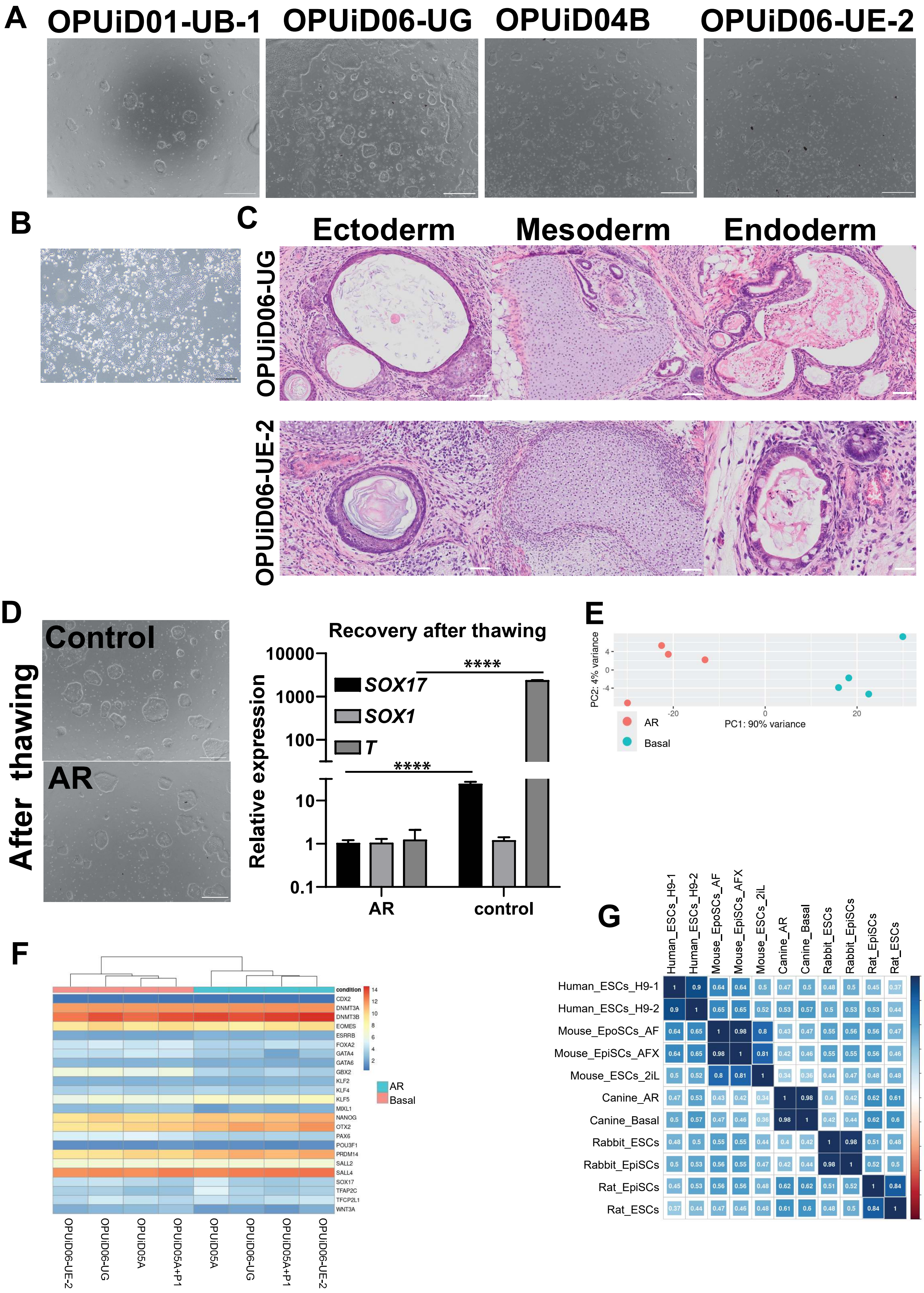

Supplemental figure 4

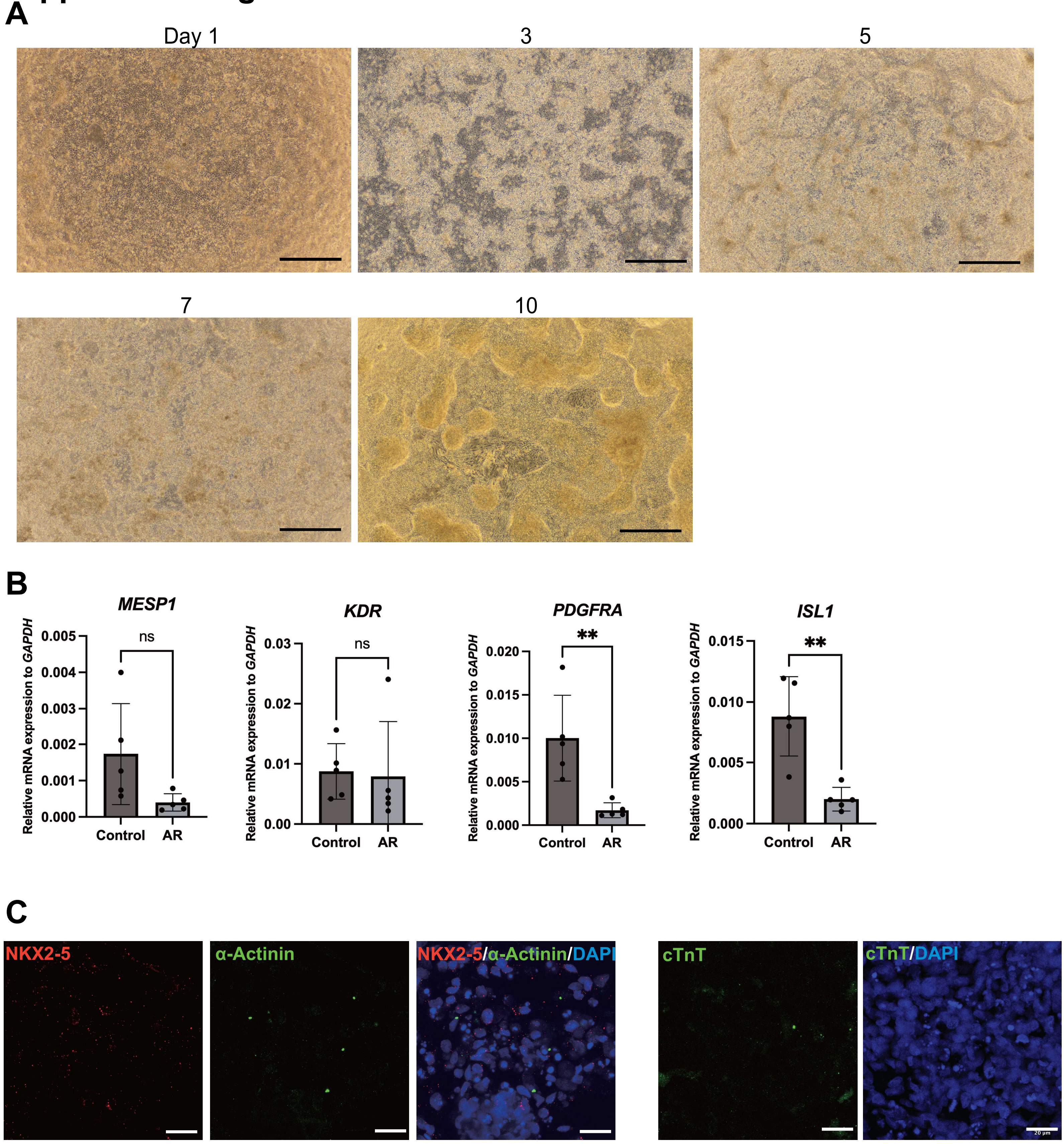

Supplementary Figure 1

(A) The representative images of ciPSCs, in culture (left), with 10 µM Y-27632 (right). Scale bar 500 µm.

(B) Quantitative RT-PCR results of the representative genes in ciPSCs in culture (left), and with 10 µM Y-27632 (right) (mean ± SD of five replicates, independent experiments).

(C) The representative electrophoresis image for RT-PCR products of *GAPDH* and *NANOG* derived from wild-type ciPSCs and NANOG-reporter cell lines (No.6, 8, 10, 11, 13, 14, and 15).

Supplementary Figure 2

(A) The frequency of $ciPSC_{high}$ cells in the NANOG-reporter ciPSC line (No.10, and 15) cultured with inhibitors (mean ± SD of three replicates, independent experiments).

(B) The frequency of $ciPSC_{high}$ cells in the NANOG-reporter ciPSC line cultured with IWP-2 or IWR-1 at different concentrations (mean ± SD of six replicates, independent experiments).

(C) The frequency of $ciPSC_{high}$ cells in the NANOG-reporter ciPSC line cultured with 500 nM IWP-2 + 10 µM IWR-1 (mean ± SD of six replicates, independent experiments).

Supplementary Figure 3

(A) The representative images of different ciPSC lines, in control ciPSC or AR medium. Scale bar 500 µm.

(B) The representative images of ciPSCs, in StemFlex based AR medium on vitronectin. Scale bar 500 µm.

(C) Representative images of hematoxylin eosin staining on the teratomas derived from different ciPSC lines cultured in AR medium. Scale bar 500 µm

(D) The representative images of ciPSCs, in control ciPSC or AR medium after thawing. Scale bar 500 µm. Quantitative RT-PCR results of the representative gene expressions in ciPSCs cultured in control ciPSC and AR medium just after recovering from frozen stock (mean ± SD of five replicates, independent experiments).

(E) PCA analysis for ciPSC lines cultured in either control ciPSC or AR medium.

(F) Gene expression pattern of ciPSC lines cultured in either control ciPSC or AR medium.

(G) Volcano plot analysis of pluripotent and lineages associated genes for ciPSC lines cultured in either control ciPSC or AR medium.

(H) Correlation matrix analysis using datasets of different species.

Supplementary Figure 4

(A) The representative images of the differentiated cells from ciPSCs in control medium at each time point. Scale bar 500 μm.

(B) Quantitative RT-PCR results of the representative gene expressions in differentiated cells on day 7 derived from ciPSCs cultured in the control and AR medium (mean ± SD of five replicates, independent experiments).

(C) Representative IF images of NKX2-5, α-Actinin, and cTnT in the differentiated cells on day 12 derived from ciPSCs cultured in the control medium. Scale bar 20 μm.

Video S1: The synchronized contraction of ciPSC-CM_{AR} at differentiation day 10
